# Supplementary material for: Structural and functional analysis of target recognition by the lymphocyte adaptor protein LNK
Source: Nat Commun. 2021 Oct 20;12:6110. doi: 10.1038/s41467-021-26394-6 (PMC8528861; doi:10.1038/s41467-021-26394-6)
Supplement: Supplementary file 1 — Supplementary Information [file 41467_2021_26394_MOESM1_ESM.pdf]

Supplementary information

Supplementary tables

Supplementary table 1. Data collection and refinement statistics for the LNK SH2 domains with phosphopeptides bound.

	**WT LNK SH2 + JAK2 pY813 (PDB ID: 7R8W)**	**WT LNK SH2 + EPOR pY454 (PDB ID:7R8X)**
**Data Collection**		
Space group	P 1 2_1 1	P 6_1
Resolution (Å)	38.59-1.9<br>(2.02-1.90)	43.08-2.3<br>(2.44-2.30)
Cell dimensions<br>a, b, c (Å)<br>α, β, γ (°)	 35.06 37.87 38.88<br>90 97.02 90	 86.16 86.16 38.05<br>90 90 120
R_{meas}	0.0424 (0.465)	0.0456 (0.539)
I /sigma(I)	18.70 (2.84)	31.00 (2.81)
$CC_{1/2}$ (%)	99.90 (86.30)	100 (90.40)
Completeness (%)	99.62 (98.88)	99.8 (99.45)
Redundancy	3.4 (3.4)	9.6 (6.1)
**Refinement**		
Resolution (Å)	38.59-1.90<br>(2.02-1.90)	33.90-2.30<br>(2.48-2.30)
No. reflections	8090 (798)	7306 (721)
R-work	19.55 (23.5)	19.62 (28.4)
R-free	25.0 (29.2)	22.95 (30.4)
No. atoms		
protein	898	923
solvent	25	13
B factors		
protein	38.64	54.51
solvent	39.93	53.79
r.m.s. deviations		
RMS(bonds)	0.009	0.004
RMS(angles)	0.92	0.65
Ramachandran favored (%)	93.81	93.81
Ramachandran allowed (%)	5.31	6.19
Ramachandran outliers (%)	0.88	0
Rotamer outliers (%)	1.14	4.40

Supplementary Table 2. Oligonucleotide sequences

Primer	Sequence
**WT mouse LNK SH2 forward**	CGCGGATCCcagaaaacagatcacttcctatcc
**WT mouse LNK SH2 reverse**	TAAGAATGCGGCCGCTAggtgttggaggaacctggtgc
**V402M human LNK forward**	AAGTTGAAAGTGAGCATGTATTCCCCACGCCGC
**R415C human LNK reverse**	GCGGCGTGGGGAATACATGCTCACTTTCAACTT
**R415C human LNK forward**	AGCCAAGCACCTGTGCCTGTCGCTGAC
**R415C human LNK reverse**	GTCAGCGACAGGCACAGGTGCTTGGCT)
**R415H human LNK forward**	GCCAAGCACCTGCACCTGTCGCTGACA
**R415H human LNK reverse**	TGTCAGCGACAGGTGCAGGTGCTTGGC

Supplementary Figures

Supplementary Figure 1- Sequence alignment and conservation of mouse and human LNK SH2 domains. Sequence alignment of the H. sapiens and M. musculus LNK SH2 domains and structure of the M. musculus LNK SH2 domain with residues differing between mouse and human highlighted in yellow.

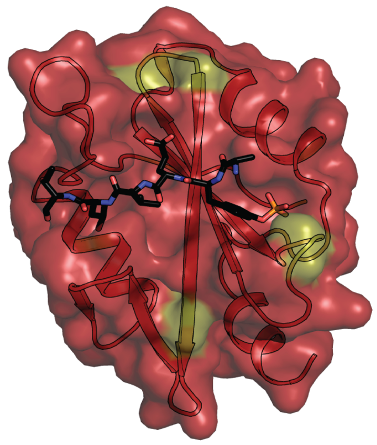

```
H. sapiens   WFHGPISRVKAAQLVQLQGPDAHGVFLVRQSETRRGEYVLTFNFQGIAKHLRLSLTERGQ
M. musculus  WFHGPISRVRAAQLVQLQGPDAHGVFLVRQSESRRGEYVLTFNLQGRAKHLRLVLTERGQ
             *********:**********************:**********:** ****** ******

H. sapiens   CRVQHLHFPSVVDMLHHFQRSPIPLECGAACDVRLSSYV 462
M. musculus  CRVQHLHFPSVVDMLRHFQRSPIPLECGAACDVRLSGYV 434
             ***************:********************.**
```

Supplementary Figure 2- Alignment of residues preceding the core LNK SH2 domain across species. a) Sequence alignment of residues within LNK from several species based on the N-terminal helix of the M. musculus SH2 domain. **b)** Structure of the LNK SH2 domain N-terminal helix positioned over a hydrophobic surface on the SH2 domain surface and alignment with N-terminal residues of SH2B and APS structures highlighting the conserved LSxYP motif**.**

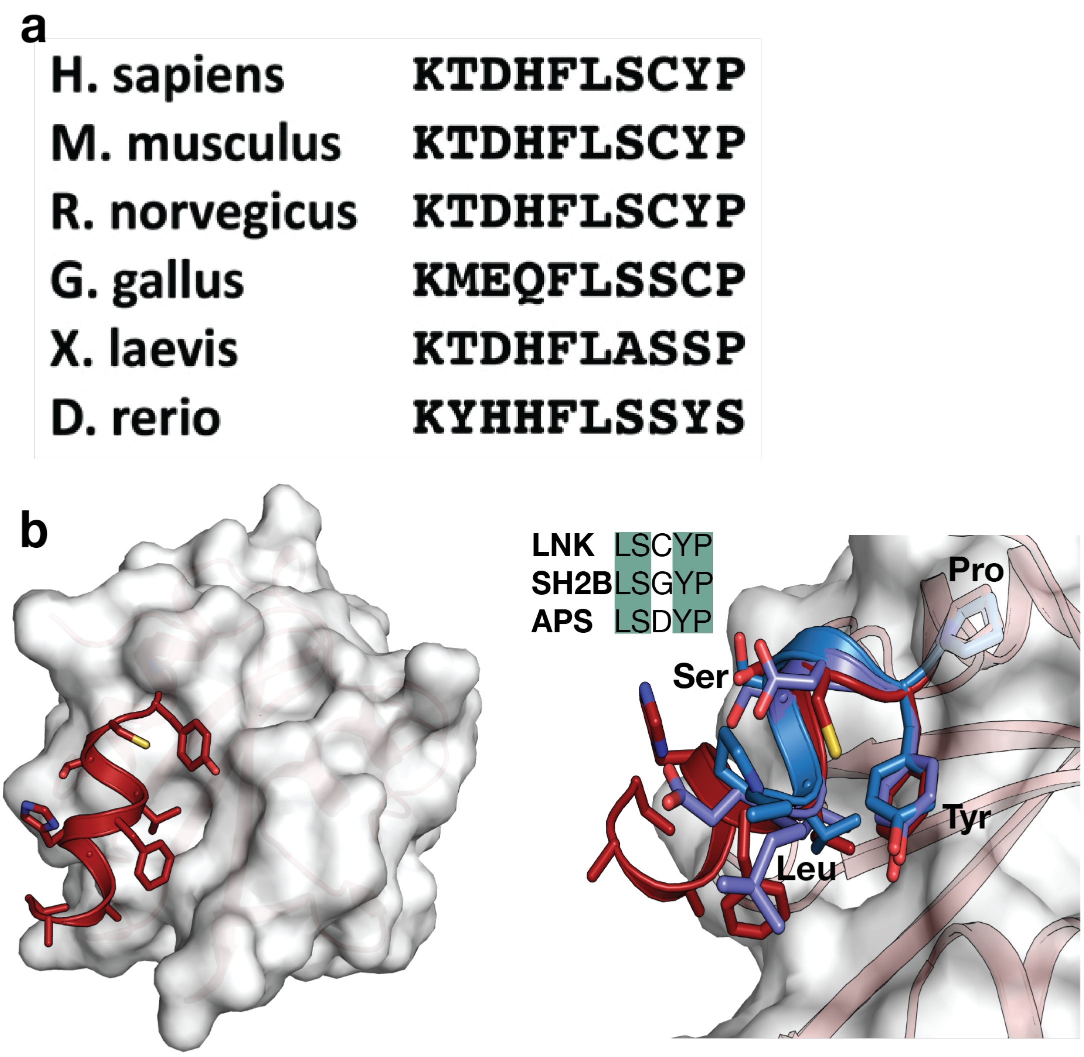

Supplementary Figure 3- Interactions between WT LNK and JAK2 pY813. 2D representation of interactions between the WT LNK SH2 domain and JAK2 pY813. Interactions are indicated by black dotted lines, with the distance between the residues indicated in angstroms. Dotted lines represent hydrogen bonds or electrostatic interactions. Red dotted lines represent salt-bridges. Visualized using LigPlot+ [1].

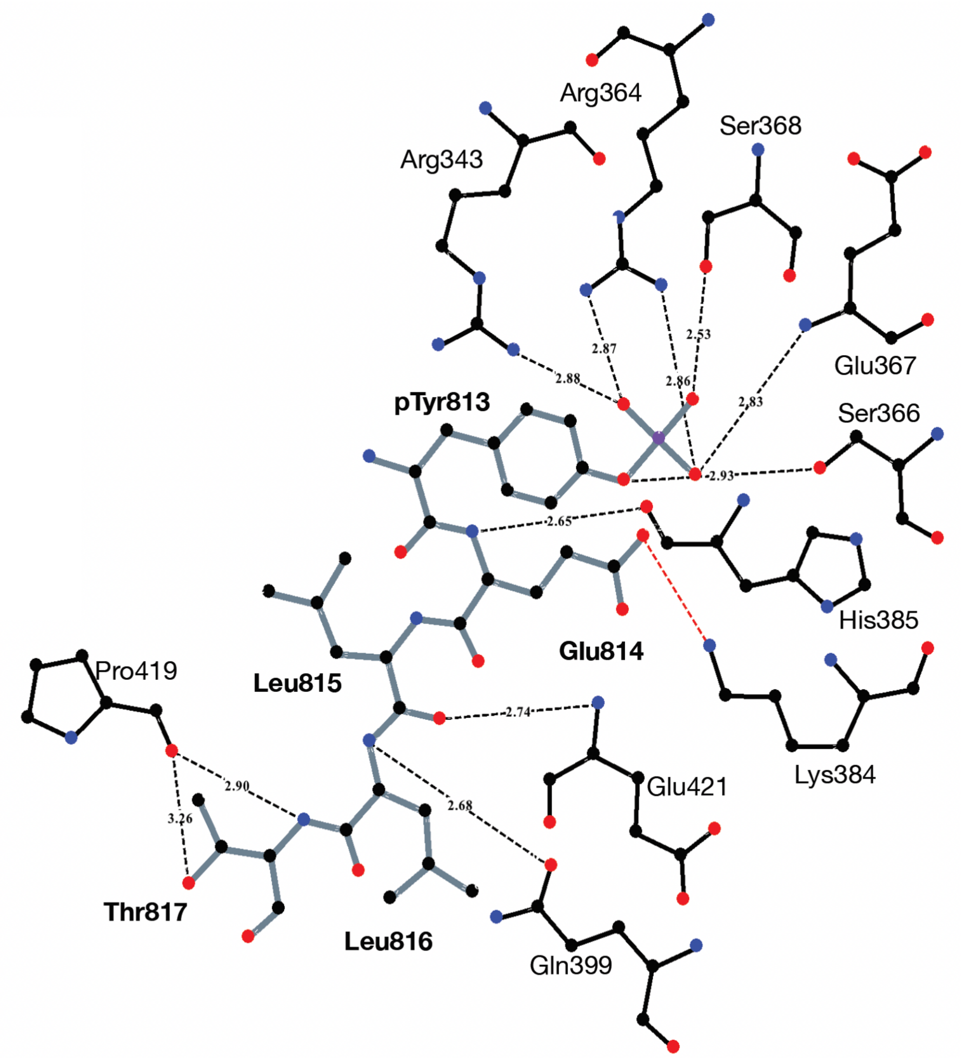

Supplementary Figure 4- Conservation of amino acid sequence across LNK, SH2B and APS SH2 domains. **a)** Structure of the M. musculus LNK SH2 domain with residues differing in APS highlighted in purple and structures of APS (PDB ID: 1RQQ) and LNK SH2 domains interacting with their binding partners (JAK2 pY813 for LNK and IRK activation loop for APS). **b)** Structure of the M. musculus LNK SH2 domain with residues differing in SH2B highlighted in blue and structures of SH2B (PDB ID: 2HDX) and LNK SH2 domains, highlighting the interactions between the SH2 domains and the JAK2 pY813 phosphopeptide.

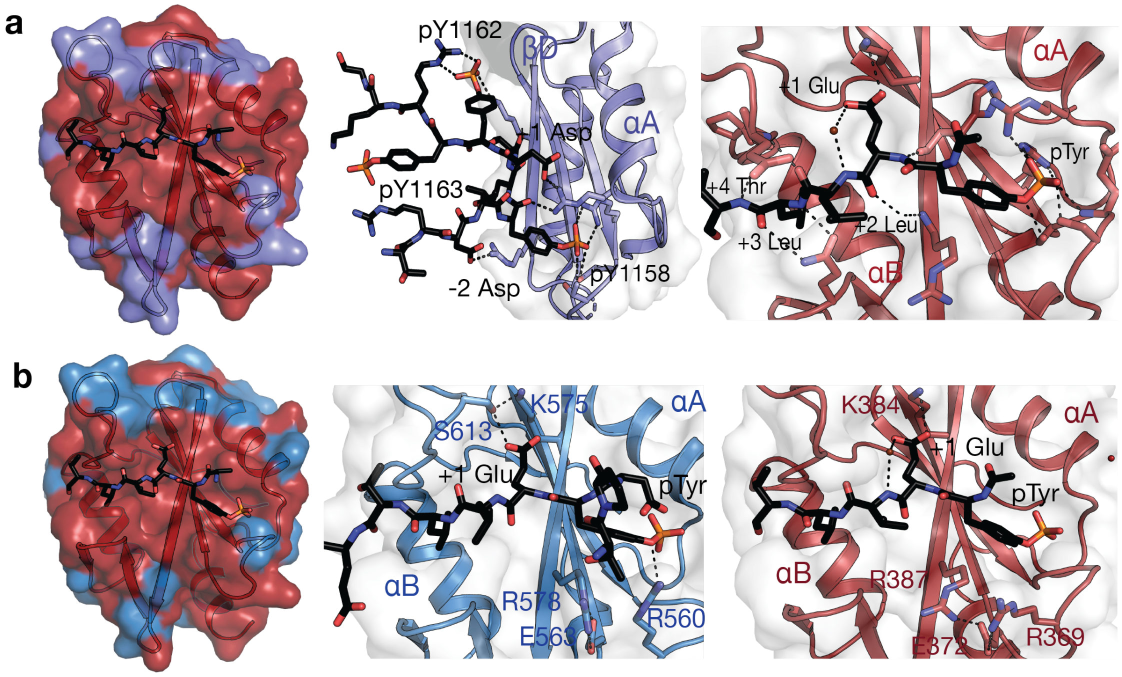

Supplementary Figure 5- Interactions between WT LNK and EPOR pY454. 2D representation of interactions between the WT LNK SH2 domain and EPOR pY454. Interactions are indicated by black dotted lines, with the distance between the residue indicated in angstroms. Dotted lines represent hydrogen bonds or electrostatic interactions. Red dotted lines represent salt-bridges. Visualized using LigPlot+ [1].

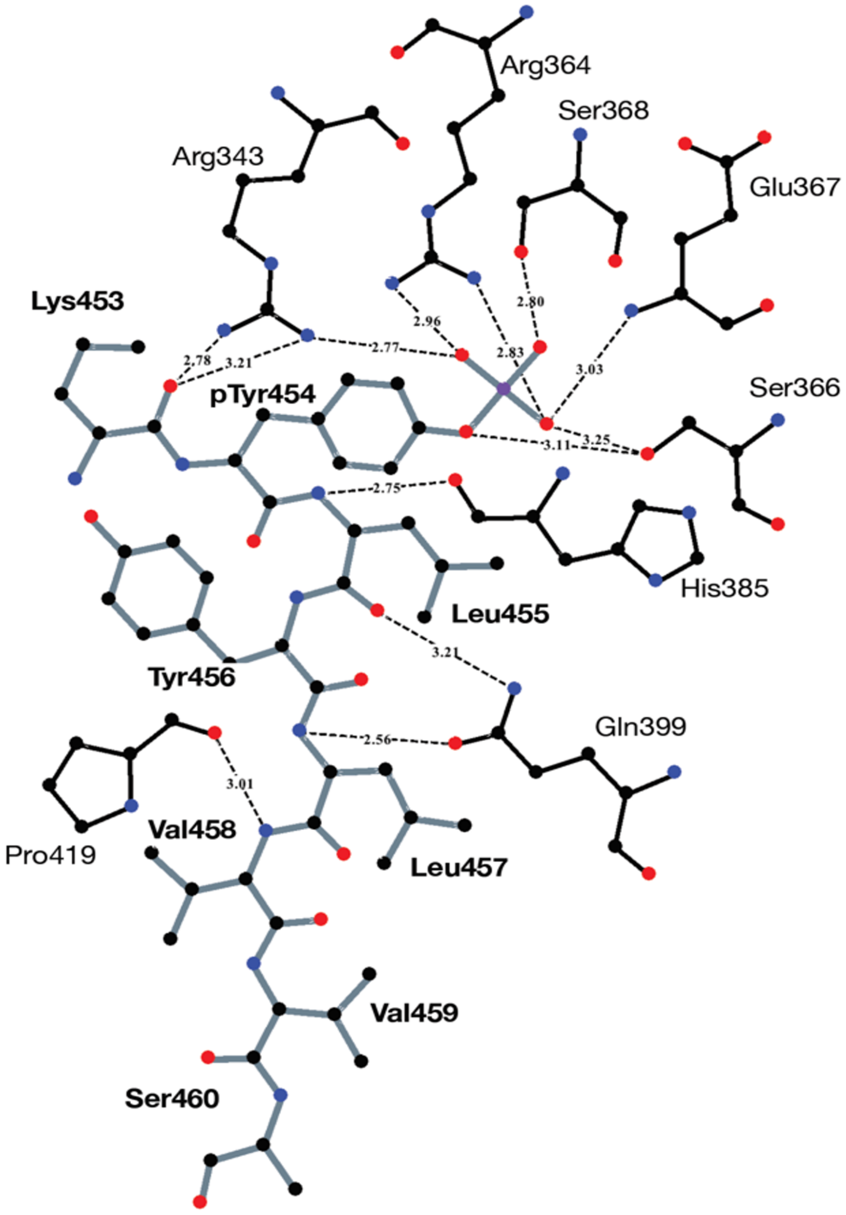

Supplementary Figure 6- Mutations within the LNK SH2 domain. R387 and V374 position within the LNK SH2 domain.

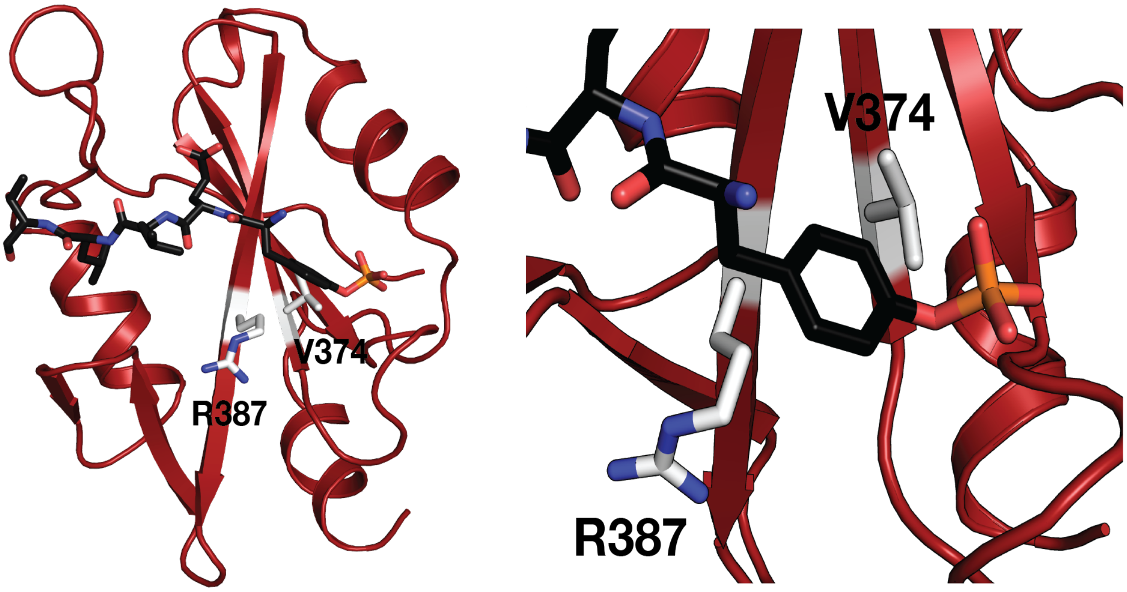

Supplementary Figure 7- LNK SH2 domain mutants bind to pY813. Representative direct binding curves for the WT, V374M, R387C and R387H LNK SH2 domains, and the SH2B SH2 domain to a JAK2 pY813 peptide. Curves represent protein concentrations as follows: 1 μM, 0.5 μM, 0.25 μM, 0.125 μM and 0.0625 μM for LNK SH2 domains and 1 μM, 0.5 μM, 0.25 μM, 0.125 μM, 0.0625 μM, 0.0315 μM, and 0.0156 μM for SH2B SH2 domain. Data are representative of at least n=2 independent experiments (n=2 for V402 and n=3 for all other SH2 domains).

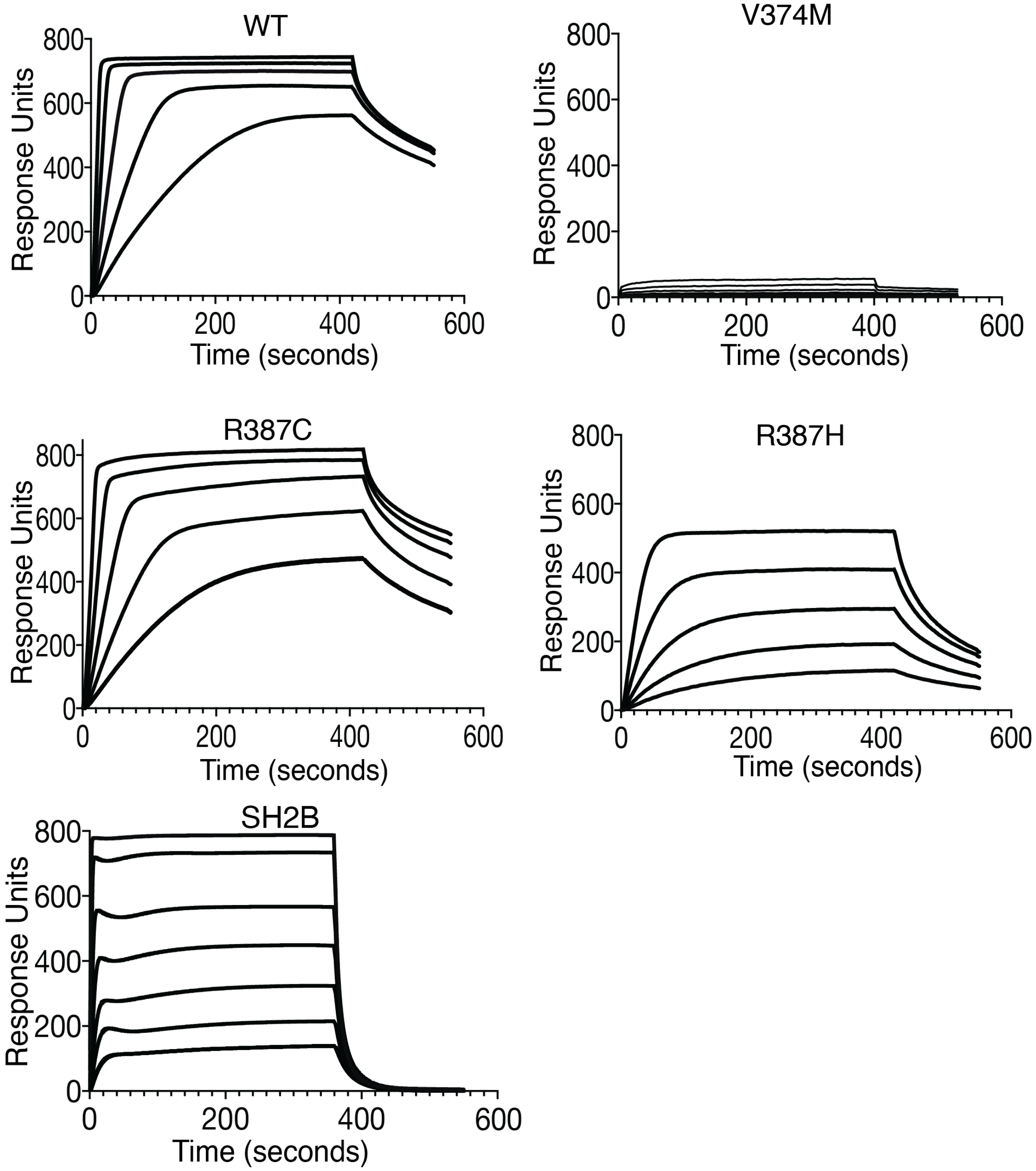
WT
V374M
R387C
R387H
SH2B
Response Units
Time (seconds)
0
200
400
600
800

Supplementary Figure 8- Thermostability increase of LNK SH2 domains in presence of 10 mM phosphotyrosine. Melting temperature (Tm) increase in °C for LNK SH2 domains in the presence of phosphotyrosine, relative to their apo Tm. Data are shown as mean ± SD of 5 technical replicates generated from n=2 independent experiments.

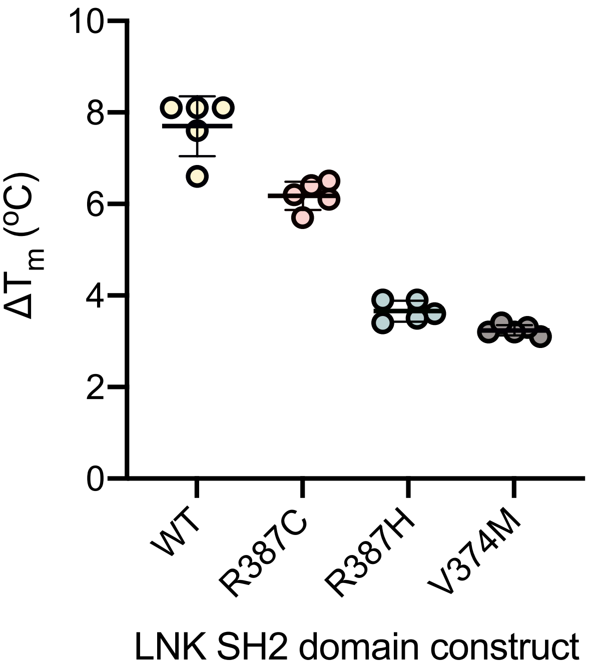

Supplementary Figure 9- Density for the BC loop of the LNK SH2 domain (JAK2 pY813 structure), JAK2 pY813 and EPOR pY454 peptides in crystal structures. a) Representation of 2Fo - Fc electron density maps of BC loop of the WT SH2/JAK2 pY813 structure, **b)** JAK2 pY813 peptide and **c)** EPOR pY454 peptide contoured at 1.5σ.

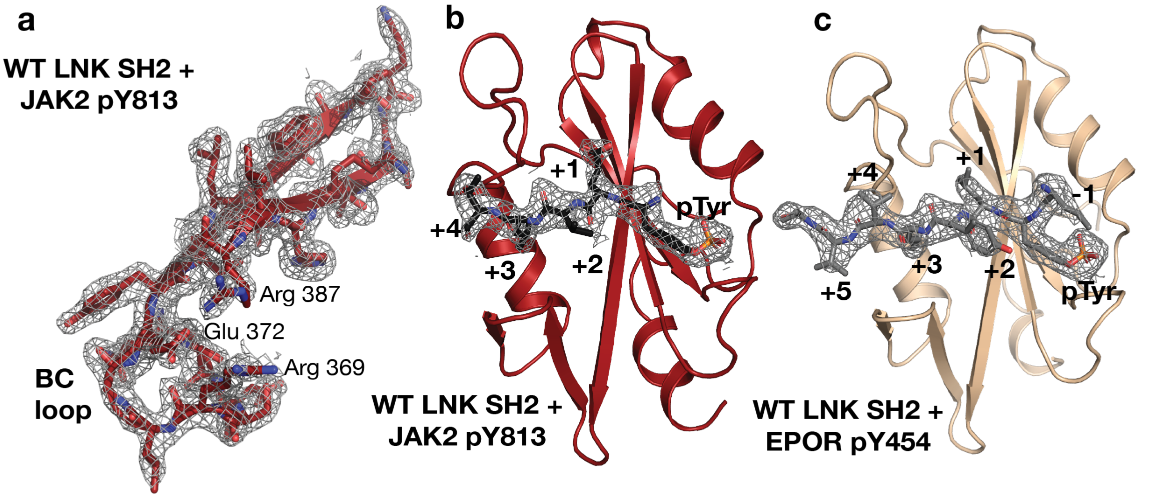

Supplementary references

1. Laskowski RA, Swindells MB. LigPlot+: multiple ligand–protein interaction diagrams for drug discovery.). ACS Publications (2011).
