# Supplementary material for: Structural and functional analysis of target recognition by the lymphocyte adaptor protein LNK
Source: Nat Commun. 2021 Oct 20;12:6110. doi: 10.1038/s41467-021-26394-6 (PMC8528861; doi:10.1038/s41467-021-26394-6)
Supplement: Supplementary file 3 — Reporting Summary [file 41467_2021_26394_MOESM3_ESM.pdf]

nature portfolio

Corresponding author(s): Jeffrey J. Babon and Nadia J. Kershaw

Last updated by author(s): 2021/09/20

Reporting Summary

Nature Portfolio wishes to improve the reproducibility of the work that we publish. This form provides structure for consistency and transparency in reporting. For further information on Nature Portfolio policies, see our Editorial Policies and the Editorial Policy Checklist.

Statistics

For all statistical analyses, confirm that the following items are present in the figure legend, table legend, main text, or Methods section.

n/a	Confirmed	
☐	☒	The exact sample size (*n*) for each experimental group/condition, given as a discrete number and unit of measurement
☐	☒	A statement on whether measurements were taken from distinct samples or whether the same sample was measured repeatedly
☐	☒	The statistical test(s) used AND whether they are one- or two-sided<br>*Only common tests should be described solely by name; describe more complex techniques in the Methods section.*
☐	☒	A description of all covariates tested
☐	☒	A description of any assumptions or corrections, such as tests of normality and adjustment for multiple comparisons
☐	☒	A full description of the statistical parameters including central tendency (e.g. means) or other basic estimates (e.g. regression coefficient) AND variation (e.g. standard deviation) or associated estimates of uncertainty (e.g. confidence intervals)
☐	☒	For null hypothesis testing, the test statistic (e.g. F, t, r) with confidence intervals, effect sizes, degrees of freedom and P value noted<br>*Give P values as exact values whenever suitable.*
☒	☐	For Bayesian analysis, information on the choice of priors and Markov chain Monte Carlo settings
☒	☐	For hierarchical and complex designs, identification of the appropriate level for tests and full reporting of outcomes
☒	☐	Estimates of effect sizes (e.g. Cohen's d, Pearson's r), indicating how they were calculated

Our web collection on statistics for biologists contains articles on many of the points above.

Software and code

Policy information about availability of computer code

Data collection	BIAcore experiments were collected with the help of BIAcore control software.<br>Stability assay data were collected using a Tycho NT.6 Nanotemper.
Data analysis	X-ray crystallography data were processed using XDS (Version Nov 1, 2016; BUILT =2016120S). Phenix 1.19.2, phaser-MR was used for molecular replacement and phenix.refine was used for refinement. Coot version 0.9 was used for model building along with Coot as part of CCP2 version 7.1. LigPlot + V2.2 was used to visualize interactions between peptide and LNK SH2 domains. PyMOL V2.3.4 was used to generate structural figures.<br>BIAcore experiments were evaluated using BIAcore Insight evaluation software. Graphpad prism 9.0 was used to further analyze BIAcore data and determine IC50 and KD values.<br>Stability assay data were collected and analysed using a Tycho NT.6 Nanotemper. Graphpad prism 9.0 was used to further analyze data stability assays. Graphpad prism 9.0 was used to analyze cellular assays and lmerTest package (Kuznetsova A., Brockhoff P.B. and Christensen R.H.B. (2017)) was used in R version 4.1.1 to carry out statistical analysis.

For manuscripts utilizing custom algorithms or software that are central to the research but not yet described in published literature, software must be made available to editors and reviewers. We strongly encourage code deposition in a community repository (e.g. GitHub). See the Nature Portfolio guidelines for submitting code & software for further information.

Data

Policy information about availability of data

All manuscripts must include a data availability statement. This statement should provide the following information, where applicable:

- Accession codes, unique identifiers, or web links for publicly available datasets
- A description of any restrictions on data availability
- For clinical datasets or third party data, please ensure that the statement adheres to our policy

Atomic coordinates for WT LNK SH2 domain with the JAK2 pY813 and EPOR pY454 phosphopeptides have been deposited in the Protein Data Bank with the accession numbers PDB 7R8W and 7R8X, respectively. The PDB files for APS (1RQQ) and SH2B (2HDX) were downloaded from the Protein Data Bank to generate FIgure 2 and Supplementary Figures 2 and 4. The data used to generate Figures 3, 5, Tables 1 and 2 and supplementary Figures 7 and 8 have been made available in the source data file.

Field-specific reporting

Please select the one below that is the best fit for your research. If you are not sure, read the appropriate sections before making your selection.

☒ Life sciences ☐ Behavioural & social sciences ☐ Ecological, evolutionary & environmental sciences

For a reference copy of the document with all sections, see nature.com/documents/nr-reporting-summary-flat.pdf

Life sciences study design

All studies must disclose on these points even when the disclosure is negative.

Sample size	Sample size for each experiment was determined by minimum number required to achieve similar robust results, however for some experiments additional repeats were performed particularly for peptides of particular interest such as JAK2 pY813. For all BIAcore experiments, a minimum of two independent experiments were performed. For peptides that did not bind such as JAK2 pY1007, JAK2 pY1008, JAK2 pY1007/1008 and IRK peptides two replicates, each from an independent experiment, was sufficient to show no binding. For all other experiments 2, 3 or 4 independent experiments were performed as follows: 2 independent experiments with 3 technical replicates from each were performed for PDGFR, c-KIT and c-FMS, 2 independent experiments with 2 technical replicates from each were performed for JAK2 pY813 mutant peptides, apart from WT JAK2 peptide in these experiments, for which 3 independent experiments were performed with a total of 5 datasets analyzed, 3 independent experiments for all FLT3, MPL and EPOR peptides was performed however for EPOR pY426 technical errors occurred and one dataset was omitted. For both JAK2 pY813 and JAK3 pY785, four independent experiments were performed. For direct binding assays with LNK SH2 domain mutants and SH2B, between 2 and 5 independent experiments were performed as follows: For stability assays with peptides and phenyl phosphate, 3 independent experiments were performed with up to 3 technical replicates for each analyzed. The number of independent repeat experiments is stated in the figure legend. For stability assays with phosphotyrosine, 2 independent experiments were performed with 2 or 3 technical replicates from each analyzed. For cellular assays, data were pooled from three independent experiments with each data point representing the mean of triplicate replicates to assure reproducibility. The number of independent repeat experiments is stated in the figure legend.
Data exclusions	Some data points were excluded from BIAcore and stability assays due to technical errors such as a damaged flow cell, or no melting curve detected. These datasets or data points were excluded from analysis.
Replication	Stability assays and cellular assays were repeated independently at least 2-3 times, while BIAcore data was obtained for at least duplicate technical replicates to ensure reproducibility. The spread of data is shown throughout the paper through use of both individual data points along with SD or SEM error bars.
Randomization	Randomization was not relevant to our study as experiments performed were quantitative not qualitative. Our study compared binding of different peptides to the LNK SH2 domain and effect of mutation on function. Human bias is mitigated in these experiments by collection and analysis by computer software predominantly.
Blinding	Blinding was not relevant to our study as experiments performed were quantitative not qualitative. Our study compared binding of different peptides to the LNK SH2 domain and effect of mutation on function. Human bias is mitigated in these experiments by collection and analysis by computer software predominantly.

Reporting for specific materials, systems and methods

We require information from authors about some types of materials, experimental systems and methods used in many studies. Here, indicate whether each material, system or method listed is relevant to your study. If you are not sure if a list item applies to your research, read the appropriate section before selecting a response.

Materials & experimental systems

n/a	Involved in the study
☒	☐ Antibodies
☐	☒ Eukaryotic cell lines
☒	☐ Palaeontology and archaeology
☒	☐ Animals and other organisms
☒	☐ Human research participants
☒	☐ Clinical data
☒	☐ Dual use research of concern

Methods

n/a	Involved in the study
☒	☐ ChIP-seq
☒	☐ Flow cytometry
☒	☐ MRI-based neuroimaging

Eukaryotic cell lines

Policy information about cell lines

Cell line source(s)	HEK293 cells sourced originally from ATCC.
Authentication	Cell lines were not genetically authenticated, however their morphology was visually confirmed.
Mycoplasma contamination	PCR and Invivogen PlasmiTest Kit determined cell lines were mycoplasma negative.
Commonly misidentified lines (See ICLAC register)	No commonly misidentified lines were used in this study.
